# The Potential of hsa-mir-106b-5p as Liquid Biomarker in Prostate Cancer Patients in Indonesia

**DOI:** 10.31557/APJCP.2021.22.3.837

**Published:** 2021-03

**Authors:** Christin H Bonnu, Anggia N Ramadhani, Ranu B Saputro, Salsabila L. Sesotyosari, R Danarto, Indwiani Astuti, Sofia M Haryana

**Affiliations:** 1 *Department of Biotechnology, Graduate School, Universitas Gadjah Mada, Yogyakarta, Indonesia.*; 2 *Department of Biomedical Science* ^*.*^ *, Faculty of Medicine, Public Health and Nursing, Universitas Gadjah Mada, Indonesia. *; 3 *Department of Urology, Faculty of Medicine, Universitas Gadjah Mada, Yogyakarta, Indonesia. *; 4 *Department of Pharmacology and Therapy, Faculty of Medicine, Universitas Gadjah Mada, Yogyakarta, Indonesia. *; 5 *Department of Histology, Faculty of Medicine, Universitas Gadjah Mada, Yogyakarta, Indonesia. *

**Keywords:** hsa-mir-106b-5p, prostate cancer, nanoString, qRT-PCR

## Abstract

**Purpose:**

This study aims to explore the potential of hsa-mir-106b-5p as a new liquid biomarker for prostate cancer sufferers in Indonesia.

**Methods:**

Analysis of hsa-mir-106b-5p expression of two tissue samples from BPH patients and two PCa patients used NanoString nCounter Expression Assay then validated by qRT-PCR using 10 patient urine samples for prostate cancer and BPH. Furthermore, analysis of the role of hsa-mir-106b-5p in prostate cancer was carried out bioinformatically.

**Results:**

The results of this study indicated that the expression of hsa-mir-106b-5p in prostate cancer tissue was 1.23 times higher than that of BPH and urine of Indonesian patients (1.72 times). Moreover, this miRNA was upregulated in prostate cancer cells compared to normal cells 1.37 times. The hsa-mir-106b-5p appeared to be involved in the development of prostate cancer through the binding of genes involved in endoplasmic reticulum stress pathways and tumor suppressor genes.

**Conclusion:**

hsa-mir-106b-5p could modulate prostate cancer by interfering with the endoplasmic reticulum stress repair pathways and decreasing the expression of tumor suppressor genes involved in many biological processes. These updates our understanding of the role of hsa-mir-106b-5p in cancer and its potential as a candidate of a biomarker for clinical diagnosis of prostate cancer.

## Introduction

Prostate cancer is cancer with the fourth-highest number of new cases in the world (7.1%). In Indonesia, the number of new prostate cancer cases until 2018 reached 11,361 cases with a mortality rate of 2.81% (GLOBOCAN, 2018). This mortality rate can be reduced if this cancer can be detected early so that the development of cancer can be prevented. The diagnosis of prostate cancer based upon the PSA level used as the main biomarker of prostate cancer is still controversial because it can provide a false positive result (Pentyala et al., 2016), while the use of a biopsy carries the risk of infectious complications (Wu et al., 2018), and can increase the risk of disease complications in patients with negative emotions (Wade et al., 2013).

Therefore, supporting diagnostic methods are required to provide a more accurate diagnosis. A liquid biopsy such as urine can be a good option because it is noninvasive, simple, and can be repeated. In the body fluids, there are various cell and tissue products, including exosomes and extracellular nanovesicles which function as a medium of communication between cells. Exosomes enclose genetic materials such as DNA, RNA, micro-RNA (miRNA), and proteins. miRNA is one of the potential genetic material in exosomes which can be developed into cancer biomarkers. These short nucleotides regulate gene expression in the body. miRNA in exosomes is stable and specific for certain cells and tissues whose expression can alter due to various conditions, including when cancer occurs (Wang et al., 2019). hsa-mir-106b-5p is a miRNA commonly known as oncomir (miRNA that is upregulated in cancer) or miRNA that binds to Tumor Suppressor Gene (TSG). Therefore, it is necessary to boost the role of hsa-mir-106b-5p contained in urinary exosomes for prostate cancer biomarkers from liquid biopsy to be more efficient and noninvasive.

## Materials and Methods

The materials used in this study were tissue and urine samples of patients with prostate cancer and BPH.

*nCounter Expression Assay*

A total of four samples consisting of two BPH samples and two prostate cancer samples were prepared in the form of FFPE at the Faculty of Medicine, Public Health and Nursing Universitas Gadjah Mada, Indonesia. The miRNA profiles of the samples were then evaluated using nCounter miRNA Assay from nanoString.

*qRT-PCR*

The urine of the ten BPH samples and ten samples of Indonesian prostate cancer patients were obtained in <3 h. The exosome isolation was then prepared using QIAGEN exosomal Kit. miRNA total was isolated using the QIAGEN miRNA Isolation kit. The cDNA was then synthesized from miRNA using the QIAGEN cDNA kit. Finally, miRNA expression was evaluated using the qRT-PCR Exiqon kit, and miRNA relative expression was calculated using the Livak method (Livak and Schmittgen, 2001).

*Bioinformatic analysis*

Prediction of signaling pathways involving miRNA was performed using the DIANA tools, evaluation of miRNA targets using STarMIr, gene interaction with miRNA using STRINGDB and Cytoscape, and prediction of prognosis using Python analysis automatically. (the data cases were downloaded from GDC Data Portal https://portal.gdc.cancer.gov/).

## Results

*Upregulated hsa-mir-106b-5p in tissue and urine samples*

Two tissue samples of PCa and BPH analyzed by nCounter expression test showed a significant difference in expression for 6 types of miRNA including hsa-mir-106b-5p by 1.23 times for prostate cancer compared to BPH (p <0.05). To validate the results of the expression analysis based on the nCounter expression test, the expression analysis was performed using the qRT-PCR method. Meanwhile, the results of the relative expression analysis with qRT-PCR based on the Livak method indicated an increase in miRNA upregulation 1.72 times higher than that of BPH. In accordance with this study, the results of the Microarray datasheet analysis (Lefort et al., 2018) also exhibited increased regulation of hsa-mir-106b-5p in prostate cancer cells compared to normal cells by 1.37 (Table 1).

*hsa-mir-106b-5p is associated with endoplasmic reticulum stress*

**Figure 1:**
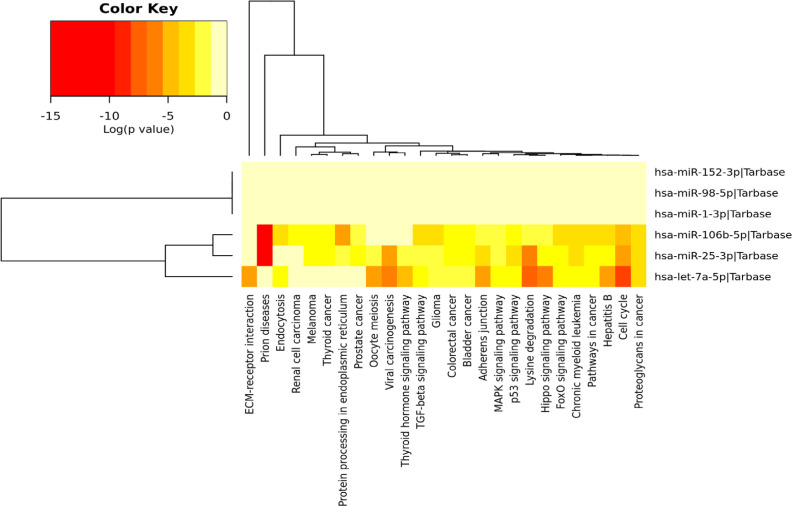
Signaling pathways involving hsa-mir-106b-5p hsa-mir-106b-5p (p <0.05).

Based on the results of the KEGG, hsa-mir-106b-5p could be involved in the processing of protein in the endoplasmic reticulum (Figure 1). There were 39 genes targeted of miRNA in this pathway. Of all genes, two genes that were the direct targets of miRNA were RAD23B and JNK with the predicted values of required energy were -20 kcal/mol and -19.8 kcal/mol, respectively (Table 2).

*Other targeted genes of hsa-mir-106b-5p*

**Figure 2:**
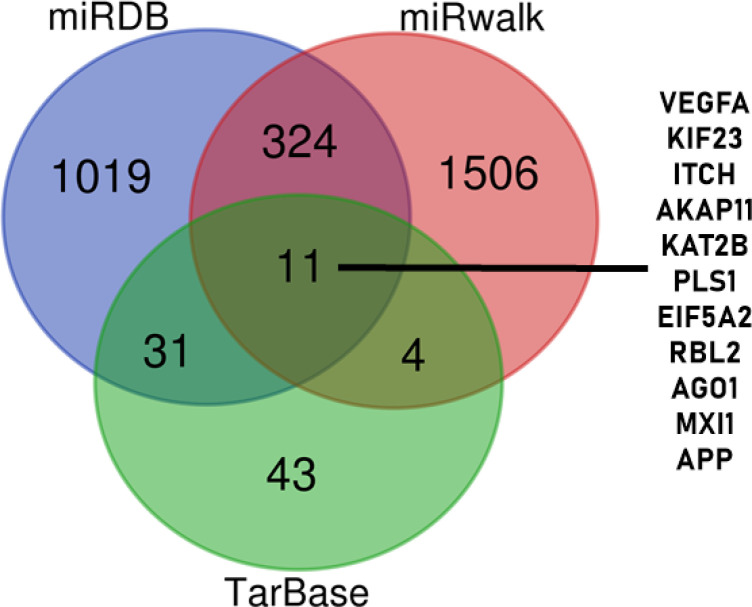
The Targeted Genes by Hsa-Mir-106b-5p Based on Bioinformatic Analysis

**Figure 3:**
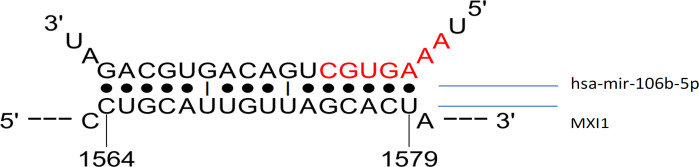
Predicted hybrid Conformation between hsa-mir-106b-5p and MXI1

**Figure 4:**
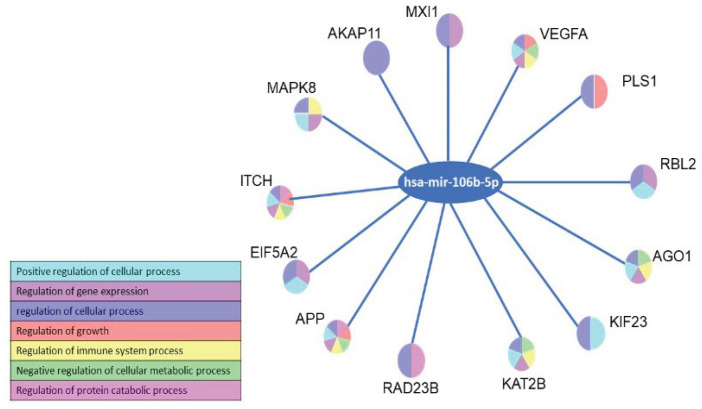
Involvement of hsa-mir-106b-5p Targeted Genes in Biological Processes

The hsa-mir-106b-5p also bind to various other genes based on analysis of miRDB (1019 genes), miRWalk (1506 genes), and TarBase (43 genes) (Figure 2). However, only 11 genes appeared simultaneously in the analysis of the three methods, namely VEGFA, KIF23, ITCH, AKAP11, KAT2B, PLS1, EIF5A2, RBL2, AGO1, MXI1, and APP (Table 2). And the MXI1 is the tergeted gene of hsa-mir-106b-5p with the minimum energy (Figure 3). All these gene are involved in many biological processes such as regulation of cellular process, gene expression, growth, immune system, cellular metabolic and protein catabolic process (Figure 4).

**Table 1:** hsa-mir-106b-5p Expression Conducted by Three Different Methods

Samples	Source	Metodhs	Fold change
PCa vs BPH	Tissue	nCounter Expression Assay	1.23
PCa vs BPH	Urine exosome	qRT-PCR	1.72
Pca vs Normal	Cell culture	Microarray	1.37

*, Tissue samples (two samples of BPH samples and two samples of Pca samples) collected from Faculty of Medicine, Public Health and Nursing Universitas Gadjah Mada; **, Urine samples are collected from BPH and PCa patients from Yogyakarta, Indonesia; ***, Microarray data were obtained from Lefort, et al (2018) then analysed bioinformatically

**Table 2:** Predicted hsa-mir-106b-5p Bond Energies with the Targeted Genes

Targeted genes	∆G (kcal/mol)
*VEGFA*	-21,4
*KIF23*	-19,1
*ITCH*	-25,1
*AKAP11*	-27,2
*KAT2B*	-26,7
*PLS1*	-23,7
*EIF5A2*	-25,2
*RBL2*	-22,6
*AGO1*	-32,3
*MXI1*	-32,6
*APP*	-22,9

* All these data obtained from Sfold (https://sfold.wadsworth.org/starmirDB.php)

**Figure 5:**
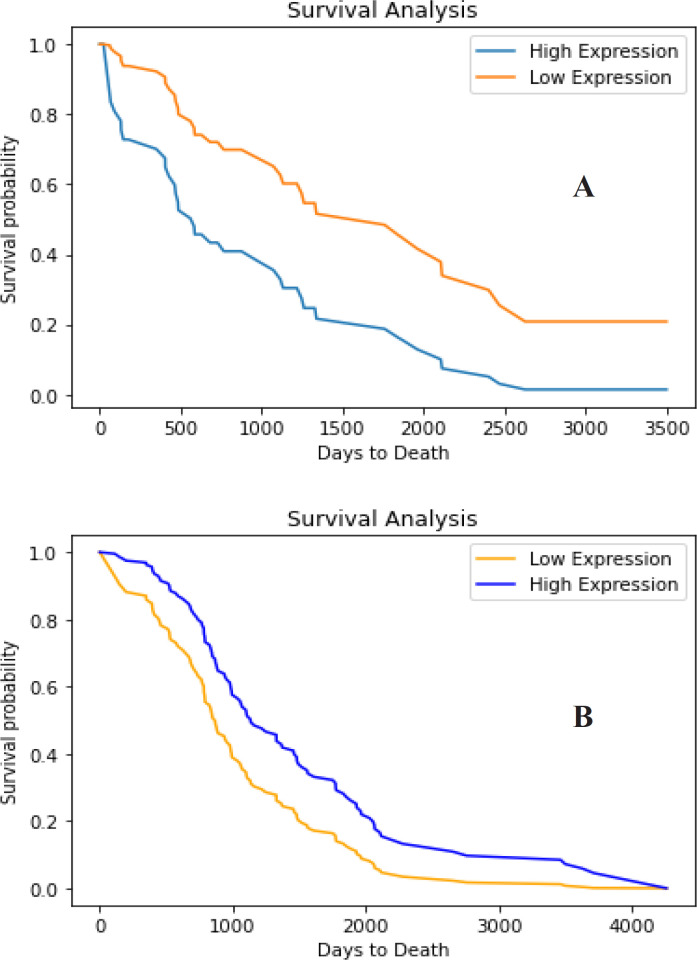
Survival Analysis of A. hsa-miR-106b-5p; B. MXI1 (p<0,05). A, Survival analysis of hsa-mir-106b-5p show poor prognosis from patient with high expression of that miRNA; B, Survival analysis of MXI1 show that patient with high expression of this gene have higher survival probability then they with lower expression

## Discussion

In this study, for the first time, we found a significant upregulation of miRNA 106b-5p in tissue samples of prostate cancer patients in Indonesia. The increase in hsa-mir-106b-5p expression based on the results of the nCounter expression test, qRT-PCR, and microarray analysis showed the consistency of miRNA expression values based on three different methods in prostate cancer compared to BPH and healthy controls. The increase in miRNA expression indicates that hsa-mir-106b-5p acts as an oncogenic mir (oncomir), miRNA that can bind to the target genes that act as tumor suppressors so that excessive miRNA expression will cause a decrease in the expression of tumor suppressor genes that support cancer development. This is supported by Goto et al., (2015) who classified hsa-mir-106b-5p as an oncomir in prostate cancer and concluded that overexpression of miRNA is associated with prostate cancer oncogenesis through downregulation of tumor suppressor genes.The most important thing that can be learned from the results of this study is that urine can be used as a noninvasive source of cancer biomarkers because the information provided is similar to the information in the tissue. Therefore, excessive tissue biopsy can be avoided.

The protein folding process does not always work perfectly, resulting in the protein failing to fold and accumulate in the endoplasmic reticulum (ER Stress). This ER stress will be responded to through a mechanism called UPR (Unfolded Protein Response) which is useful to restore proteostasis so that cells can survive (Limonta et al., 2019). The UPR mechanism will fail to restore proteostasis if the stress-triggering factors of ER have exceeded the tolerance capacity of UPR which then requires UPR to shift cell fate from survival to apoptosis. Cells that continue to grow in cancer cases result in a decrease in the supply of cell nutrients and an increase in Reactive Oxygen Species (ROS) continuously, as well as cause ER stress. ER stress triggers UPR activation which leads to apoptosis because cells cannot tolerate the accumulation of ROS. The mechanism of apoptosis triggered by ER stress is mediated by IRE1α, PERK, and ATF6 (Kara and Oztas, 2019).

As an oncomir that supports cancer development, it is closely related to the effect caused by miRNA when it binds to genes that have a complementary base sequence with it. This binding will cause inhibition of target gene translation so that the target gene will have two possible fates: inhibition or degradation. The stronger the bond that occurs between this miRNA and the target gene, the more genes will be degraded. hsa-mir-106b-5p might contribute to cancer progression by inhibiting the apoptosis mediated by ER stress and inhibiting the action of TSG.

The binding of MAPK8 pro-apoptosis by hsa-mir-106b-5p occurs through the IRE1α (IRE1) pathway. UPR activation in prostate cancer through the IRE1α pathway begins with the recruitment of TRAF2 by IRE1α which causes the activation of ASK1. ASK1 will then phosphorylate JNK resulting in BCL2 inhibition and increased expression of BAX and BAK in mitochondria. These two genes will then cause the release of Cyt-C from the mitochondria which will trigger the activation of Caspase 3/7 that leads to apoptosis. This is in accordance with the research of Ma et al., (2018) that found ASK1-JNK activation could lead to apoptosis mediated by mitochondria in prostate cancer.

RAD23B is the hsa-mir-106b-5p target associated with resistance mechanisms toward UV therapy (Hatano et al., 2014). Linge et al. also reported that downregulation of this gene could lead to more invasive cancer due to its role as a tumor suppressor gene in cancer. However, the inhibition mechanism of this gene by hsa-mir-106b-5p which can support cancer development still requires further investigation.

MXI1 is a gene that functions in the regulation of gene transcription. Based on the results of bioinformatic analysis, the bond between hsa-mir-106b-5p and this gene requires the least amount of energy. MXI1 with a minimum binding energy value has a hsa-mir-106b-5p attachment location of 140 of the total length of the 228 bp gene. This allows inhibition of genes by miRNA and even degradation. MXI1 negatively controls the activity of Myc oncoprotein so that it can function as a tumor suppressor in prostate cancer. Downregulation of this gene occurs in 50% of prostate cancer cases and is predicted to contribute to the pathogenesis of cancer (Yan et al., 2018). However, in contrast to the results of this study, Kuczyk et al., (1998) found that this gene does not affect prostate cancer formation at an early stage but is upregulated due to the downregulation of PTEN as a tumor suppressor.

In this study, although there is no confirmation of gene-target interactions by in vitro or in vivo methods, reviewing the results of bioinformatic analyzes and previous studies, we suggest that down-regulated MXI1 plays pivotal role during prostate cancer development. This is supported by the poor prognosis of hsa-mir-106b-5p in prostate cancer patients. Conversely, the Kaplan Meier MXI1 chart shows that patients with higher gene expression has a longer survival time than patients with lower gene expression (Figure 5). Therefore, further analysis is required regarding the effect of this gene expression on prostate cancer progression.

In addition, hsa-mir-106b-5p also targets genes that play an important role in immunity such as APP, VEGFA, KAT2B, MAPK8, and AGO1 and this miR-mRNA binding allows post-transcription disruption of these genes to influence cancer development. Simultaneously, the results of this study indicate the contribution of hsa-mir-106b-5p to the aggressiveness of prostate cancer.

In summary, hsa-mir-106b-5p has the potential to become a biomarker of prostate cancer through regulation of various genes involved in various biological processes, so that further studies of this research are expected to comprehend the mechanism of miRNA regulation in prostate cancer.

## Author Contribution Statement

Study conception and design: Sofia M. Haryana, Indwiani Astuti, R.Danarto. Data collection: Christin H. Bonnu, Ranu B. Saputro, Anggia N. Ramadhani, Salsabila L. Sesotyosari, R. Danarto. Analysis and interpretation of result: Christin H. Bonnu. Draft manuscript preparation: Christin H. Bonnu, Sofia M. Haryana, Indwiani Astuti
